# Clinical and Autoimmune Profile of Scleroderma Patients from Western India

**DOI:** 10.1155/2014/983781

**Published:** 2014-10-19

**Authors:** Vandana Pradhan, Anjali Rajadhyaksha, Milind Nadkar, Pallavi Pandit, Prathamesh Surve, Maxime Lecerf, Jagadeesh Bayry, Srinivas Kaveri, Kanjaksha Ghosh

**Affiliations:** ^1^National Institute of Immunohaematology, Parel, Mumbai 400012, India; ^2^Department of Medicine, King Edward Memorial Hospital, Parel, Mumbai 400012, India; ^3^Institut National de la Santé et de la Recherche Médicale Unité 1138, Centre de Recherche des Cordeliers, UPMC Univ Paris 06, 75006 Paris 6, France

## Abstract

*Background*. Systemic sclerosis (SSc, scleroderma) is a disorder characterized by fibrosis of skin and visceral organs. Pathogenesis of scleroderma is complex and is incompletely understood as yet. Autoantibodies in SSc represent a serologic hallmark which have clinical relevance, with diagnostic and prognostic potential. *Objectives*. To study distribution of clinical manifestations and to identify frequency of autoantibodies among subtypes of scleroderma patients from Western India. *Methodology*. One hundred and ten scleroderma patients were clinically classified according to the American College of Rheumatology/European League Against Rheumatism (ACR/EULAR) criteria. All these patients were in active stage of disease. Clinical manifestations were recorded at the time of presentation. Autoantibodies were tested in them by indirect immunofluorescence test and ELISA. Immunoglobulin levels were estimated by nephelometer. These parameters were further correlated with clinical presentation of the disease. *Results*. Scleroderma patients had M : F ratio of 1 : 10 where mean age at evaluation was 34.7 ± 10.7 years and a mean disease duration was 43.7 ± 35 months. Clinical subtypes showed that 45 patients (40.9%) had diffused cutaneous (dcSSc) lesions, 32 patients (29.1%) had limited cutaneous (lcSSc) lesions, and 33 patients (30%) had other autoimmune overlaps. The overall frequency of ANA in SSc patients studied was 85.5%. The frequency of anti-Scl70, anti-centromere, anti-endothelial cell antibodies (AECA), and anti-keratinocyte antibodies (AKA) was 62.7%, 22.7%, 30%, and 40.9%, respectively. Anti-Scl70 antibodies were significantly high (75.6% versus 46.9%) among dcSSc patients (*P* < 0.0115) whereas anti-centromere antibodies were significantly high (9% versus 38%) among lcSSc patients when these two subtypes were compared (*P* < 0.0044). *Conclusion*. This study supports that there are geoepidemiological variations among scleroderma patients for their clinical presentation, autoantibody profile, and immune parameters across the country.

## 1. Introduction

Scleroderma or systemic sclerosis (SSc) is a complex autoimmune disease affecting 1/100,000 individuals among the Caucasian population. The prevalence rate of this disease is around 5/100,000 with an incidence of 1/100,000. Higher rates have been reported in USA, Australia, and Eastern Europe and lower rates have been reported in Northern Europe and Japan [1]. Even though current clinical and diagnostic utilities have led to a better understanding of the disease, its pathogenesis still remains unknown. Scleroderma is a heterogeneous disease with a wide range of clinical manifestations ranging from mild skin fibrosis with minimal internal organ disease to severe skin and organ involvement. The three main pathological events that are involved in scleroderma pathogenesis are mainly endothelial damage, fibrosis, and autoimmune dysregulation. Etiopathogenesis of scleroderma is characterized by fibroproliferative alterations, cellular and humoral immune abnormalities resulting in a severe and often progressive fibrotic process [2].

Scleroderma can also be subdivided according to different criteria, such as involvement of organs and the presence of specific antibodies which are hallmarks of the disease. These autoantibodies are disease-specific and usually mutually exclusive and correlate with the extent of skin involvement and associated disease manifestations. The most common are DNA topoisomerase (anti-Scl70), anti-centromere antibodies (CENP A and/or B protein). These autoantibodies are marker antibodies for relatively distinct clinical phenotypes of SSc where anti-Scl70 antibodies are a marker for dcSSc and SSc patients with clinically significant pulmonary fibrosis with a poor prognosis whereas anti-centromere antibodies typically are associated with lcSSc, uncommon pulmonary fibrosis, and late onset of pulmonary hypertension but generally are associated with an overall good prognosis [3, 4].

Geoepidemiologically it has been noted that clinical features and presence of these disease-specific autoantibodies vary across the globe and ethnicities [5]. The low incidence of SSc and the clinical variability result in difficulties in understanding the disease pathogenesis. There is an unmet need for validated biomarkers for scleroderma disease diagnosis, classification, and future therapeutic approach for management of scleroderma patients. This study was designed to look at differences in the clinical features among subset of scleroderma patients with an emphasis on autoantibodies in scleroderma patients from Mumbai, Western India.

## 2. Material and Methods

### 2.1. Study Design

This prospective study was conducted in 110 scleroderma patients from Rheumatology Department of King Edward Memorial Hospital, Mumbai, India, and National Institute of Immunohaematology, Mumbai, India, over the period of 3 years (2010–2012).

### 2.2. Ethics Statement

This study was carried out after obtaining the requisite ethics committee approval and a written consent from patients.

### 2.3. Clinical Classification

Scleroderma patients were classified according to American College of Rheumatology/European League Against Rheumatism (ACR/EULAR) criteria [6, 7]. Clinical features at the time of evaluation were recorded in proformae. SSc patients were classified into two clinical subgroups based on the extent of skin involvement, limited cutaneous SSc (lcSSc), and diffuse cutaneous SSc (dcSSc) that are associated with different clinical complications and prognoses. Diffused cutaneous (dcSSc) patients had delayed Raynaud's phenomenon, severe constitutional symptoms, arthralgias, carpal tunnel, puffy hands and legs, palpated tendon friction rubs, skin thickening progressing from fingers to trunk rapidly, potentially severe pulmonary fibrosis, and cardiac and renal involvement. lcSSc patients had Raynaud's phenomenon alone for years, rate constitutional symptoms, minimal arthralgias, puffy fingers, telangiectasias and late calcinosis, skin thickening limited to hands and face, and mild pulmonary fibrosis. Patients with no skin thickening were also included in lcSSc group. Severe organ system involvement noted like musculoskeletal involved joints/tendons of fingertip to palm distance 4.0+ cm and severe proximal muscle weakness on physical examination. Severe renal manifestations involved “renal crisis” with serum creatinine 3.0+ mg/dL at any time. Severe gastrointestinal tract manifestations involved malabsorption syndrome and episodes of pseudoobstruction. Pulmonary arterial hypertension was diagnosed by right-sided heart catheterization according to standard definitions. Pulmonary fibrosis was seen on chest radiography and Raynaud's phenomenon was self-reported or reported in patients with at least a 2-phase colour change in finger(s) and often toes consisting of pallor, cyanosis, and/or reactive hyperemia in response to cold exposure. Pregnant and postmenopausal women, smokers, patients with diabetes, and patients with significant hyperlipemia were excluded.

### 2.4. Methodology

Standard investigations like CBC, ESR, routine biochemical tests (renal and liver function tests and electrolytes), chest X ray, and ECG were carried for all patients. Special investigations such as endoscopy and biopsies were performed as per the requirement. Standard investigations included CBC. After blood collection, sera were stored in aliquots at −80°C until being tested. Anti-nuclear antibodies (ANA) (BioRad, USA), anti-endothelial cell antibodies (AECA) (Euroimmune, Germany), and anti-keratinocyte antibodies (AKA) (Euroimmune, Germany) were tested by indirect immunofluorescence test (IIF). Anti-Scl70 antibodies (anti-I), anti-centromere antibodies, and Anti-cyclic citrullinated peptide (CCP) antibodies were tested by ELISA using commercially available kits (Euroimmune, Germany).

### 2.5. Statistical Analysis

Continuous variables were expressed as mean ± SD. Pairs of groups were compared using Student's test for normally distributed continuous distribution. The “*χ* ^2^” test was used for the categorical variables as needed. Statistical significance was set at *P* < 0.05.

## 3. Results

A total of 110 patients with scleroderma having mean age of 34.7 ± 10.7 years at evaluation and a mean disease duration of 43.7 ± 35.4 years were included in the study (Table 1). The starting point for calculation of disease duration was at the disease onset. There were 100 females (91%) and 10 males (9%) included in this study. The female to male ratio was 10 : 1. At evaluation women were slightly older than males with mean ± SD of 35.6 ± 10.5 as compared to males (30.7 ± 5.7). The disease duration ranged between 6 and 120 months with a mean ± SD of 43.7 ± 35.4.

It was observed that 45 patients (40.9%) had dcSSc lesions and 32 patients (29.1%) had lcSSc lesions. The remaining 33 patients (30%) had other autoimmune overlaps wherein patients had features of scleroderma combined with features of a second connective tissue disease at the time evaluation. Clinical presentation revealed that 108 patients (98.2%) had cutaneous manifestations, 85 patients (77.3%) had pulmonary manifestations, 12 patients (10.9%) had renal involvement, 43 patients (39.1%) had musculoskeletal involvement, 8 patients (7.3%) had gastrointestinal involvement, and 15 patients (13.5%) had cardiovascular involvement.

As shown in Table 2, the overall frequency of ANA in SSc patients studied was 85.5%. It was observed that 60 patients (63.9%) had speckled pattern, 17 patients (18.1%) had nucleolar pattern, 7 patients (7.4%) had centromere pattern, 2 patients (2.1%) had rim/peripheral pattern, and the remaining 2 patients (2.1%) had speckled and nucleolar pattern. The frequency of anti-Scl70 antibodies, anti-centromere antibodies, AECA, and AKA was 62.7%, 22.7%, 30%, and 40.9%, respectively. For anti-Scl70 antibodies among dcSSc compared with lcSSc there was a statistically significant difference (*P* < 0.0115, OR = 3.5030, and 95% CI = 1.3256–9.2570). When anti-centromere antibodies were compared in both the groups, there was a statistically significant difference noted (*P* < 0.0044, OR = 0.1626, and 95% CI = 0.0465–0.5684). When dcSSc and lcSSc patients were compared statistically, there was no statistically significant difference for ANA, AECA, and AKA (*P* > 0.05). When dcSSc and lcSSc patients together were compared with overlap patients, no statistically significant difference was noted for autoantibodies (*P* > 0.05).  Table 3 gives an association of these autoantibodies with organ manifestations in SSc patients.

## 4. Discussion

Geoepidemiology studies suggest that systemic sclerosis (SSc) is more common, occurs only at a younger age, and is more severe in African Americans than Caucasians. The major differences noted were mainly in their clinical and serological phenotypes. These differences can be further related to different environmental exposure as well as immunogenetic makeup of these patients. The studies on clinical and serological profile of SSc patients have important implications for both clinical interventions and future pathogenic studies [8]. de Souza Müller et al. had documented 65.62%, 26.04%, and 8.33% frequency for dcSSc, lcSSc, and overlap among their total SSc patients from Brazil [3]. Wielosz et al. had reported 74% GI manifestations among SSc patients from Poland [9]. Indian data on SSc patients revealed that, among North and South Indian SSc patients, the clinical presentation varies [10, 11]. Though there are a few published records from Western India there is no information available from the Eastern part of the country giving details of clinical profile of Indian SSc patients. Demographic and serological similarities/differences in Indian scleroderma patients in various regions across the country are as shown in Table 4 [10–15].

SSc-related autoantibodies among scleroderma patients were compared in different ethnicities like Caucasian Americans, African Americans, and Latin Americans by Krzyszczak et al. [5]. The reported frequency of anti-Scl70 and anti-centromere antibodies was 15%, 17%, respectively, in Caucasian American, 35%, 0% in African American, and 20%, 40% among Latin American SSc patients [5]. Mierau et al. had reported an incidence of 94.2% ANA positivity among German SSc patients and among Brazilian SSc patients, 92.4% ANA positivity had been reported [4, 16]. Present study showed a slightly lower incidence for ANA. There are only a few reports on frequency of autoantibodies in Indian SSc patients. Johnson et al. had reported a much lower incidence (17%) for anti-Scl70 autoantibodies among Canadian SSc patients, as compared to 62.7% in the present study whereas the incidence for anti-centromere antibodies was 29% which was similar to the present study (22.7%). 19%, 21% anti-Scl70 antibodies and anti-centromere antibodies from Pittsburg, USA, 17%, 16% from Toronto, 35%, 27% from Madrid, and 22%, 28% from Berlin SSc cohort had been documented [17]. Among Brazilian SSc patients anti-Scl70 frequency reported was 17.8% which was mainly associated with dcSSc (*P* < 0.015) whereas anti-centromere antibodies (33.3%) were commonly associated with lcSSc subtypes [4]. Mierau et al. had reported a total incidence of 30.1% for anti-Scl70 antibodies where dcSSc subtype was strongly associated with anti-Scl70 autoantibodies (*P* < 0.0001) [16]. Frequency of anti-centromere antibodies reported by the same group was 35.9% and a positive correlation was found in lcSSc patients with anti-centromere antibodies (*P* < 0.0001). de Souza Müller et al. also had reported a positive correlation of anti-centromere antibodies with lcSSc form (*P* < 0.01) which are similar to the present study [3].

Previous studies had shown that anti-centromere antibodies were strongly associated with renal dysfunction in lcSSc patients and CENP-B was a major target antigen reported for AECA in lcSSc patients indicating the association between anti-centromere antibodies and AECA autoantibodies leading to AECA mediated endothelial dysfunction due to an underlying autoimmune mechanism [18–21]. Further endothelial dysfunction may be associated with high incidence of cerebrocardiovascular diseases which needs to be studied in SSc patients [22]. This study throws light on a need for some biomarker antibodies and discovery of new target antigens among scleroderma patients and their immunodiagnostic potential. These disease-specific antibodies along with disease phenotype variation across the country will help in understanding the geoepidemiological picture of scleroderma in different geographical regions in India. This could possibly represent new diagnostic and/or prognostic markers of scleroderma in the near future.

## Figures and Tables

**Table 1:** Clinical presentation of scleroderma patients studied (*n* = 110).

Clinical features	Number (*n* = 110)	Percentage positivity
Cutaneous	**108**	**98.2%**
Skin thickening	80	74.1%
Peripheral vascular	**98**	**89.1%**
Raynaud's phenomenon	75	76.5%
Digital ulcers and/or gangrene	23	23.5%
Pulmonary	**85**	**77.3%**
Interstitial lung disease (ILD)	75	88.2%
Pulmonary hypertension	52	61.2%
Renal	**12**	**10.9%**
Musculoskeletal	**43**	**39.1%**
Gastrointestinal	**8**	**7.3%**
Cardiovascular	**15**	**13.6%**
Diffuse cutaneous (dcSSc)	**45**	**40.9%**
Limited cutaneous (lcSSc)	**32**	**29.1%**
Other autoimmune disease overlaps	**33**	**30%**
Rheumatoid arthritis (RA)	8	24.2%
Mixed connective tissue disease (MCTD)	5	15.2%
Systemic lupus erythematosus (SLE)	4	12.1%
Polymyositis/dermatomyositis	5	15.2%
Others (Sjogren's syndrome, and hypothyroidism)	11	33.3%

**Table 2:** Distribution of autoantibodies in subgroups of scleroderma patients (*n* = 110).

Autoantibodies *n* (%)	Diffused (*n* = 45)	Limited (*n* = 32)	Overlap (*n* = 33)
ANA 94 (85.5%)	39 (86.7%)	28 (87.5%)	27 (81.8%)
Anti-Scl70 antibodies 69 (62.7%)	34∗ (75.6%)	15 (46.9%)	20 (60.6%)
Anti-centromere antibodies 25 (22.7%)	04∗∗ (9%)	12 (38%)	09 (27%)
AECA 33 (30%)	09 (20%)	15 (48%)	09 (27%)
AKA 45 (40.9%)	18 (40%)	10 (31%)	17 (51%)

^*^ *P* < 0.0115, OR = 3.5030, and 95% CI = 1.3256–9.2570.

^**^ *P* < 0.0044, OR = 0.1626, and 95% CI = 0.0465–0.5684.

**Table 3:** Association of autoantibodies with organ manifestations in SSc patients (*n* = 110).

Autoantibodies	Pulmonary	Renal	Musculoskeletal	Gastrointestinal	Cardiovascular
ANA (*n* = 94)	65 (69.1%)	80 (85.1%)	75 (79.8%)	52 (55.3%)	40 (42.6%)
Anti-Scl70 antibodies (*n* = 69)	28 (40.6%)	40 (58%)	48 (69.6%)	10 (14.5%)	10 (14.5%)
Anti-centromere antibodies (*n* = 25)	18 (72%)	20 (80%)	08 (32%)	08 (32%)	10 (40%)
Anti-endothelial cell antibodies (AECA) (*n* = 33)	15 (45.5%)	28 (84.9%)	14 (42.4%)	10 (30.3%)	24 (72.7%)
Anti-keratinocyte antibodies (AKA) (*n* = 45)	20 (44.4%)	18 (40%)	10 (22.2%)	05 (11.1%)	05 (11.1%)

**Table 4:** Demographic and serological similarities/differences in Indian scleroderma patients in various regions across the country.

Regions of India			
	Western ∗Present, reference [15]	Northern Reference [10–12]	Southern Reference [13, 14]
Disease onset			
dsSSc	40.9%	94%, 88.1%	
IcSSc	29.1%	6%, 11.9%	
Cutaneous			
Skin thickening	98.2%		
Peripheral vascular			
Raynaud's phenomenon	68.2%, 83.3%	92.9%, 60%	17.3%, 28.2%
Digital ulcers and/or gangrene	20.9%		
Pulmonary			
Interstitial lung disease (ILD)	88.2%		
Pulmonary hypertension	61.2%		
Renal	10.9%, 8.3%	3.4%, 6%, 20%	10.3%
Musculoskeletal	39.1%, 54.2%	36.7%, 80%	66.7%
Gastrointestinal	7.3%, 50%		
Cardiovascular	13.6%, 12.5%		
Autoantibodies			
ANA	85.5%	89.1%, 70%	
Anti-Scl70	62.7%	55.5%	
Anti-centromere	22.7%		

^*^There are no published reports from Eastern part of the country.
